# Prognostic Value of Autophagy, Microsatellite Instability, and *KRAS* Mutations in Colorectal Cancer

**DOI:** 10.7150/jca.51430

**Published:** 2021-04-20

**Authors:** Yuanyuan Wang, Zhi Zhao, Jing Zhuang, Xinxin Wu, Zhizhong Wang, Bing Zhang, Ge Gao, Yinping Zhang, Caili Guo, Qingxin Xia

**Affiliations:** 1 Department of Pathology, Affiliated Cancer Hospital of Zhengzhou University, Henan Cancer Hospital, 127 Dongming Road, Zhengzhou 450008, China.; 2 Department of Pathology, Yihe Hospital, Henan University, No. 69 Agriculture East Road, Zhengzhou 450008, China.; 3 Department of General Surgery, Affiliated Cancer Hospital of Zhengzhou University, Henan Cancer Hospital, 127 Dongming Road, Zhengzhou 450008, China.; 4 Department of Molecular Pathology, Affiliated Cancer Hospital of Zhengzhou University, Henan Cancer Hospital, 127 Dongming Road, Zhengzhou 450008, China.; 5 Department of Critical Care Medicine, Affiliated Children's Hospital of Zhengzhou University, No. 255 Gangdu Road, Dongsan Street, Zhengzhou 450008, China.

**Keywords:** Beclin 1, LC3, MSI, KRAS, prognosis, CRC.

## Abstract

**Introduction:** Autophagy plays pivotal role in various tumors, including colorectal cancer (CRC). Microsatellite instability (MSI) and *KRAS* mutations are also involved in response to the adjuvant therapy of CRC. We aimed to investigate the relationships among autophagy, *KRAS* mutations, MSI, clinicopathological parameters, and prognosis in CRC patients. **Methods and Results:** We tested 200 CRC tumors for autophagy-related protein expression (Beclin 1 and LC3), MSI status, and *KRAS* mutations. **Results:** Expression of Beclin 1 and LC3 was higher in CRC, with Beclin 1 significantly correlating with the depth of invasion, whereas LC3 was not associated with clinicopathological parameters. Patients expressing the LC3 proteins experienced a shorter overall survival (OS) after surgery with adjuvant therapy, especially in the MSS/L-CRC subgroup and the mutated *KRAS* subgroup. MSS/L-CRC patients with KRAS mutations positively expressed the LC3 protein and suffered a shorter OS than LC3 non-expressing patients. In CRC patients who received either capecitabine or capecitabine combined with oxaliplatin post-surgery, the positive expression of LC3 correlated with worse OS compared to patients who did not express LC3. Sequencing showed BRCA1/2 as the most variant genes in all patients. Nevertheless, deleterious variations were more frequent in patients with MSI-H CRC. **Conclusions:** High LC3 protein expression shows a certain prognostic value in CRC patients. LC3, the MSI status, and *KRAS* mutations must be considered when selecting an adjuvant therapy for CRC. The detection of these indexes is of great significance to identify high-risk patients who would benefit from autophagy-related anticancer drugs or help to explore more effective treatment options for patients who are resistant to conventional chemotherapy or relapse.

## Introduction

Colorectal cancer (CRC) is the third most commonly diagnosed malignancy worldwide, seriously endangering human health 1. At present, treatment for CRC usually involves surgical resection combined with chemotherapy or radiation therapy. However, due to differences in the genetic background among different individuals, drug resistance remains a widely unresolved issue 2. Therefore, in recent years, achieving personalized precision medical CRC treatment has been the focus of research.

Studies have shown that several molecular mechanisms are involved in the development of CRC tumors, of which microsatellite instability (MSI) (15%) and chromosomal instability (CIN) (75%) play the most important roles 3, 4. The gold standard for MSI testing recommended by the National Cancer Institute is using the polymerase chain reaction (PCR) method to detect MSI markers (BAT25, BAT26, D2S123, D5S346, and D17S250) 5. According to the test results of MSI analysis, patients with CRC can be divided into three groups: high-frequency microsatellite instability (MSI-H) with two or more genes showing instability, low-frequency MSI (MSI-L) with only one locus showing genetic deletion, and microsatellite stable (MSS) with no gene loss.

The KRAS proto-oncogene (KRAS) is known to belong to the RAS family of proteins and has been reported to be frequently mutated in various types of tumors, including CRC 6. Activation of KRAS has been shown to depend on the receptor tyrosine kinase (RTK) MAPK/PI3K signaling pathway that has been known to promote the cellular proliferation of the tumor. The mutant *KRAS* is constitutively activated without requiring epidermal growth factor receptor (EGFR) signals, whereas the wild type *KRAS* gene requires an EGFR signal to be activated. So, KRAS mutations have been shown to directly lead to treatment failure in patients with CRC undergoing an anti-EGFR therapy, such as cetuximab 7, 8. National Comprehensive Cancer Network (NCCN) guidelines explicitly list cetuximab as a first-line treatment for KRAS wild type CRC.

Autophagy removes damaged or aged intracellular organelles and abnormal proteins, which is essential for cell homeostasis 9. Recently, autophagy has been extensively reported in various types of tumors, including breast, pulmonary, brain, prostate, and colorectal cancer. To date, due to its dual function, autophagy was considered to be a double-edged sword in carcinogenesis. The process of autophagy is known to be regulated and encoded by autophagy-related genes (ATGs), with more than 30 ATGs being identified in yeast 10, of which Beclin 1 (BECN1) and microtubule-associated protein 1A/1B-light chain 3 (LC3) are the two critical autophagy markers used in this study. The reported results on autophagy in CRC have been inconclusively conflicting, so its function in CRC development and progression remains unclear.

The guidelines of NCCN indicate that patients with stage II MSI-H CRC have a better outcome but do not benefit from fluorouracil (5-FU) adjuvant therapy 11. It has been shown that autophagy inhibition could increase 5-FU-induced apoptosis *in vitro* 12, 13. So, we speculate that autophagy might be related to the failure of MSI-H patients from 5-FU treatment. Furthermore, autophagy was induced by EGFR siRNA in cancer cells 14, and *KRAS* acts downstream of EGFR. In conclusion, the relationship between autophagy and MSI/KRAS is still unclear. In this study, the status of MSI, KRAS, and autophagy-related proteins, namely, Beclin 1 and LC3 in CRC patients were evaluated. We focused on the correlation between MSI, KRAS, and autophagy in CRC and the prognosis to provide the foundation for studying the targeted antitumor therapy.

## Materials and methods

### Patients

For this study, 200 formalin-fixed paraffin-embedded (FFEP) CRC tumor tissues were collected from the Department of Pathology, Henan Cancer Hospital (Zhengzhou, China). Control samples were derived from normal mucosal tissues ≥ 5 cm away from the tumor edge for immunohistochemistry. All patients with CRC were categorized based on the recommendations by the seventh American Joint Committee on Cancer Tumor-Node-Metastasis (TNM) staging 15. The patients with CRC consisted of 110 males and 90 females, aged 31-85 y with a median age of 60 y. None had received radiotherapy or chemotherapy prior to surgery, excluding those with genetic adenomatous polyposis and Lynch syndrome. There were 21 patients in stage I, 95 in stage II, 73 in stage III, and 11 in stage IV. Among the 200 patients, 51 patients did not receive any adjuvant therapy after surgery, 24 patients received capecitabine chemotherapy, 72 patients received capecitabine plus oxaliplatin combined chemotherapy, and 46 patients received other chemotherapy or combined radiotherapy. The postoperative information of 7 patients was unknown. All patients were enrolled from March to November 2015 following surgery. Clinical follow-up information was obtained by telephone calls from the date of the surgery until January 2021. Of the 200 patients with CRC enrolled in the present study, 51 patients did not receive adjuvant therapy after surgery; 7 patients whose postoperative information was unknown were also excluded from the survival analysis; finally, 8 cases did not complete their follow-up. In total, 72 patients survived, and 62 patients died. The OS of all patients ranged from 4 to 66 mo. The study was approved by the Institutional Ethics Committee of the Affiliated Cancer Hospital of Zhengzhou University. Signed informed consent forms were obtained from all patients or their guardians.

### Immunohistochemistry (IHC)

Tissue sections (4 µm thick) for immunohistochemistry were deparaffinized in xylene, followed by rehydration with serially decreased ethanol concentrations. Then, tissues were placed in citrate buffer (pH 6.0) for antigen retrieval (95 ºC, 15 min). Endogenous peroxidase was blocked by 3% H_2_O_2_ solution for 10 min at 25 ºC. Sections were incubated with each primary antibody for 2 h at 25 ºC, washed three times with phosphate-buffered saline (PBS), and then incubated with secondary antibodies for 20 min at 25 ºC. Subsequently, sections were incubated with DAB substrate for 5-10 min. Anti-LC3 (ab48394, 1:800) and anti-Beclin1 (ab114071, 1:450) were purchased from Abcam (Cambridge, UK). The expression of autophagy-related proteins was evaluated by measuring the percentage of positively stained cells and the staining intensity. The percentage of positively stained cells was graded as follows: 0, ≤ 5%; 1, 6-35%; 2, 36-65%; and 3, 66-100%. The staining intensity was graded as follows: 0, no staining; 1, buff; 2, yellow; and 3, brown. The final staining score was calculated by multiplying the above-obtained scores. Tumors with an immunoreactive score of 0-3 were designated as negative, whereas those with 4-9 were classified as positive. All sections were submitted to 2 pathologists for evaluation.

### Amplification refractory mutation system PCR (ARMS-PCR)

Extracted DNA from CRC paraffin blocks was subjected to PCR analysis according to the manufacturer's instructions of the human KRAS gene mutation detection kit (AmoyDx, Xiamen, China). The reaction protocol was as follows: 95 ºC, 5 min; 95 ºC, 25 s, 64 ºC, 20 s, 72 ºC, 20 s (15 cycles); 93 ºC, 25 s, 60 ºC, 35 s, 72 ºC, 20 s (31 cycles); and 72 ºC for 10 min. The FAM and HEX signals were collected under the condition of 60 ºC. The results of this study illustrate the potential use of GAPDH as a reference gene to analyze KRAS mutations.

### PCR-capillary electrophoresis

The PCR-capillary electrophoresis was recommended for the detection of MSI in CRC samples. DNA was extracted to detect MSI using 5 microsatellite sites, including *BAT25*, *BAT26*, *D2S123*, *D5S346*, and *D17S250*. The primer sequences were as follows: BAT25 forward, 5'-TCGCCTCCAAGAATGTAAGT-3' and reverse, 5'-TCTGGATTTTAACTATGGCTC-3'; BAT26 forward, 5'-TGACTACTTTTGACTTCAGCC-3' and reverse, 5'-AACCATTCAACATTTTTAACC-3'; D2S123 forward, 5'-AAACAGGATGCCTGCCTTTA-3' and reverse, 5'-GGACTTTCCACCTATGGGAC-3'; D5S346 forward, 5'-ACTCACTCTAGTGATAAATCGGG-3' and reverse, 5'-AGCAGATAAGACAAGTATTACTAG-3'; and D17S250 forward, 5'-GGAAGAATCAAATAGACAAT-3' and reverse, 5'-GCTGGCCATATATATATTTAAACC-3'. The reaction system was comprised of 20 μL, including 2 μL primers, 10 μL enzymes, 1-8 μL template DNA, and 0-7 μL deionized water. The conditions of the PCR reaction were as follows: denaturation (42 ºC, 5 min; 94 ºC, 5 min), 40 cycles (94 ºC, 15 s; 55 ºC, 25 s; 72 ºC, 50 s); and 72 ºC for 10 min. The ABI 3500XL Genetic Analyzer (Applied Biosystems, Foster City, CA, USA) was used to detect the PCR products following the manufacturer's instructions. Results were analyzed using the GeneMapper v4.1 (Applied Biosystems) software.

### Next-generation sequencing

Library construction was performed following the manufacturer's protocol of the 19 gene sequencing kit (Otogenetics, Atlanta, USA), which includes the DNA sequences of all exons of androgen receptor (*AR*), ATM serine/threonine kinase (*ATM*), BRCA1 associated RING domain 1 (*BARD1*), BRCA1 DNA repair associated (*BRCA1*), BRCA2 DNA repair associated (*BRCA2*), BRCA1 interacting protein C-terminal helicase 1 (*BRIP1*), caspase 8 (*CASP8*), cadherin 1 (*CDH1*), checkpoint kinase 2 (*CHEK2*), DIRAS family GTPase 3 (*DIRAS3*), Erb-B2 receptor tyrosine kinase 2 (*ERBB2*), nibrin (*NBN*), partner and localizer of BRCA2 (*PALB2*), phosphatase and tensin homolog (*PTEN*), RAD50 double-strand break repair protein (*RAD50*), RAD51 recombinase (*RAD51*), serine/threonine kinase 11 (*STK11*), transforming growth factor-beta 1 (*TGFB1*), and tumor protein p53 (*TP53*). Briefly, 100 μL of DNA was extracted from each sample and fragmented to prepare the DNA library by performing end-repairing, adaptor ligation, size selection (250-700 bp), and hybridization. Then, DNA samples were amplified, captured with Streptavidin Dynabeads (Otogenetics), and selected with magnetic beads followed by subsequent PCR amplification and purification using AMPure beads (Otogenetics). The final pool was used for sequencing using Illumina MiSeq sequencer (Illumina, California, USA) (250-750 bp).

### Statistical analysis

Statistical analysis was performed using the Statistical Package for the Social Science (IBM Corp., Armonk, NY, USA) version 21.0. The statistical significance of the protein expression of autophagy markers (Beclin 1 and LC3) and MSI in the tumor samples versus normal controls were compared using the χ^2^ or Fisher's exact and paired-samples *t*-tests. The statistical significance of *KRAS* mutations in the tumor versus control samples was compared using paired-samples χ^2^ test. The relationship between the protein expression of autophagy markers, MSI, as well as mutations in KRAS and clinicopathological factors were also determined by the same methods. The correlation of autophagy, MSI, and KRAS was evaluated by Spearman's rank correlation test. Kaplan-Meier and log-rank methods were used to calculate overall survival (OS) rates, and the survival curves were compared by the log-rank test. In all statistical analyses, a P-value < 0.05 was considered statistically significant.

## Results

### Beclin 1 and LC3 proteins expression in CRC

**Figure 1:**
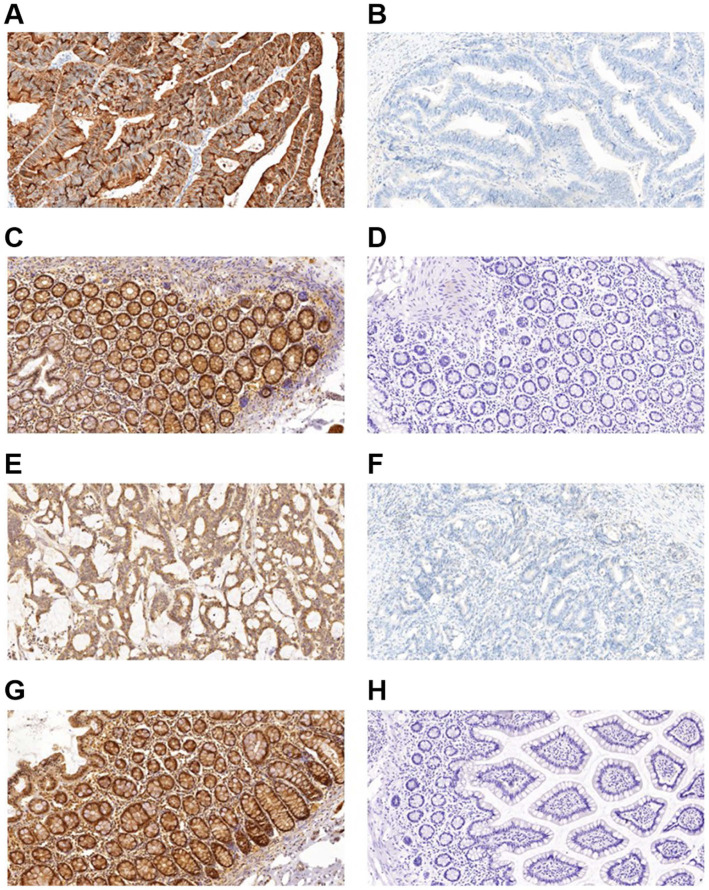
Immunohistochemical staining for autophagy-related proteins (Beclin 1 and LC3) in CRC shows positivity in the cytoplasm. Beclin 1 protein positive (score: 9) (A) and negative expression (B) in CRC; Beclin 1 protein positive (score: 9) (C) and negative expression (D) in normal intestinal mucosa; LC3 protein positive (score: 9) (E) and negative expression (F) in CRC; LC3 protein positive (score: 9) (G) and negative expression (H) in the normal intestinal mucosa. All the pictures are magnified 100×.

Figure 1 shows representative immunohistochemistry results, and the expression of autophagy-related proteins in CRC tumor cells was predominantly localized in the cytoplasm. Table 1 compared the expression of autophagy-related proteins in CRC. A paired-samples *t*-test demonstrated that autophagy-related proteins were differentially expressed between CRC tissues and controls, while both the P-values of Beclin 1 and LC3 were less than 0.001 (Table 1). The positive expression frequencies of the autophagy-related proteins were demonstrated to be 84.00% (168/200) for Beclin 1, and 86.50% (173/200) for LC3 among the 200 CRC tumor samples. In contrast, in 200 normal mucosal tissues, we observed 4 samples expressing Beclin 1 and 6 samples expressing LC3. The expression of the Beclin 1 (χ^2^ = 274.337, P < 0.001) and LC3 (χ^2^ = 281.999, P < 0.001) autophagy-related proteins was much higher in CRC tumor samples than in the intestinal mucosa group (Table 1). No significant correlation was demonstrated between the protein expression of Beclin 1 and LC3 (r = 0.067, P = 0.345).

### Association between Beclin 1 and LC3 with clinicopathologic features, and overall survival (OS) in CRC

**Figure 2:**
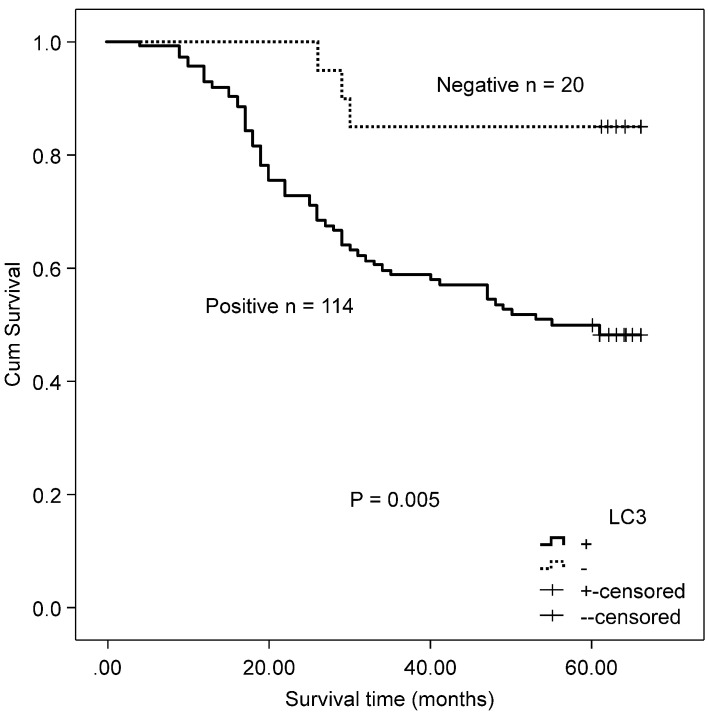
Kaplan-Meier survival curves comparing survival time for patients with CRC with LC3 expression.

The association between the autophagy-related proteins (Beclin 1 and LC3) and clinicopathologic features in CRC samples was further analyzed (Table 2). Positive expression of Beclin 1 significantly correlated with the depth of invasion (P = 0.005), whereas no significant correlation was observed between Beclin 1 expression and other clinicopathologic parameters and OS (χ^2^ = 2.846, P = 0.092) by Kaplan-Meier analysis. LC3 expression was not associated with any of the assessed clinicopathological parameters, but patients with positive expression of LC3 experienced a worse OS after surgery than those with a negative protein expression (χ^2^ = 7.917, P = 0.005, Fig. 2).

### Microsatellite instability (MSI) in CRC

In this study, PCR was used to evaluate the state of MSI in CRC samples. Accordingly, we identified 26 samples with MSI-H, one sample with low MSI-L, and 173 samples that were MSS (Supplementary Table 1). Supplementary Figure 1 shows the MSI state in samples. The frequency of MSI-H was 13% in CRC, whereas the control samples were all MSS. MSI-H was significantly different between CRC and control groups (χ^2^ = 27.807, P < 0.001; t = 5.237, P < 0.001).

### Association between MSI and clinicopathologic features, and OS in CRC

As only 1 case of MSI-L-CRC was found, MSI-L and MSS were combined into 1 group for statistical analysis. Concomitantly, we researched the relationship between the MSI-H and MSS/L groups in CRC (Table 3). We found that the MSI-H state was related to age, lymph node metastasis, location, and tumor differentiation, but not to gender, mucous adenocarcinoma, depth of invasion, and TNM stage. No association was found between MSI and OS in all patients with CRC (χ^2^ = 0.360, P = 0.548).

### Association between the autophagy-related proteins (Beclin 1 and LC3) and clinicopathologic features, and OS in colorectal cancer of the MSI subgroup

There was no significant correlation observed between autophagy-related proteins and MSI by χ^2^ test (Beclin 1: χ^2^ = 0.906, P = 0.341; LC3: χ^2^ = 0.371, P = 0.341, Table 2), and the Spearman's rank test (Beclin 1: r = 0.088, P = 0.217; LC3: r = ‑0.065, P = 0.362). Expression of both Beclin 1 and LC3 was shown to be unrelated to all clinicopathological parameters in patients with MSI-H CRC. No association was also found between autophagy-related proteins and OS in patients with MSI-H CRC (Beclin 1: χ^2^ = 0.609, P = 0.435; and LC3: χ^2^ = 1.332, P = 0.248).

**Figure 3:**
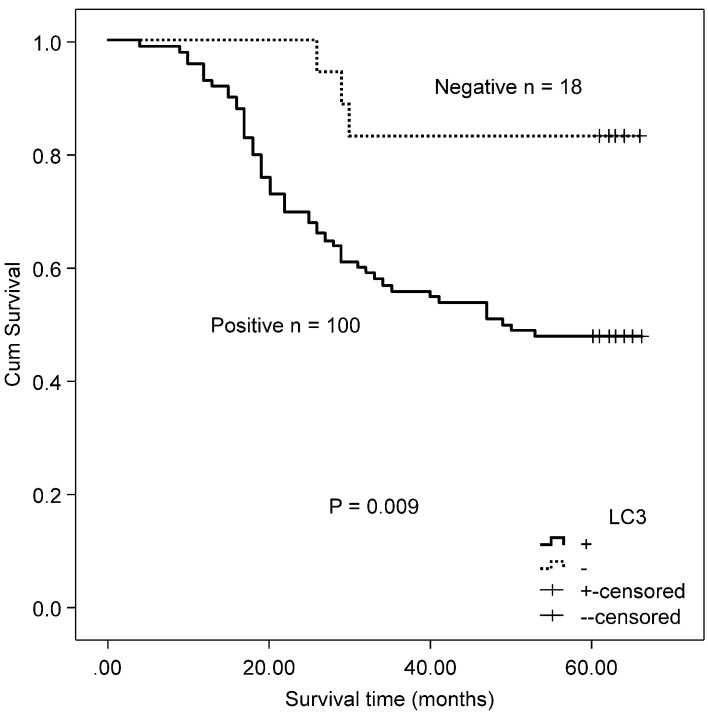
Kaplan-Meier survival curves comparing survival time for CRC patients with MSS/L expressing LC3.

In the MSS/L-CRC subgroup, the expression of Beclin 1 was demonstrated to be higher in the T3/T4 group (85.52%) compared with the T1/T2 group (68.97%) (Table 4). No association was found between Beclin 1 protein expression and OS in MSS/L-CRC patients (χ^2^ = 2.618, P = 0.106). The expression of LC3 was shown to be unrelated to all clinicopathological parameters in the MSS/L-CRC subgroup (Table 4). LC3-expressing patients in the MSS/L-CRC subgroup had a worse OS than those non-expressing LC3 (χ^2^ = 6.732, P = 0.009, Fig. 3).

### *KRAS* mutations in CRC

Following qPCR analysis, mutations in the *KRAS* gene were found in 80 of the 200 CRC samples (codon 12 in 65 cases; codon 13 in 15 cases), with a mutation rate of 40.00% (Supplementary Table 2). Supplementary Figure 2 shows *KRAS* mutation in CRC samples. All control samples were *KRAS* wild type; *KRAS* mutation rates in CRC samples were higher than in control samples (χ^2^ = 100.00, P < 0.001).

### Association between *KRAS* mutations and clinicopathologic features, and OS in CRC

In this study, the rate of *KRAS* mutations was related to gender, depth of invasion, and TNM stage (Table 3). Moreover, our findings showed no statistically significant association between mutations in the* KRAS* gene and age, lymph node metastasis, tumor location, tumor differentiation, or mucous adenocarcinoma (Table 3). No association was found between mutations in the *KRAS* gene and OS in patients with CRC (χ^2^ = 0.305, P = 0.581).

### Correlation of the autophagy-related proteins (Beclin 1 and LC3) and clinicopathologic features, and OS in colorectal cancer with mutated/wild type *KRAS*

**Figure 4:**
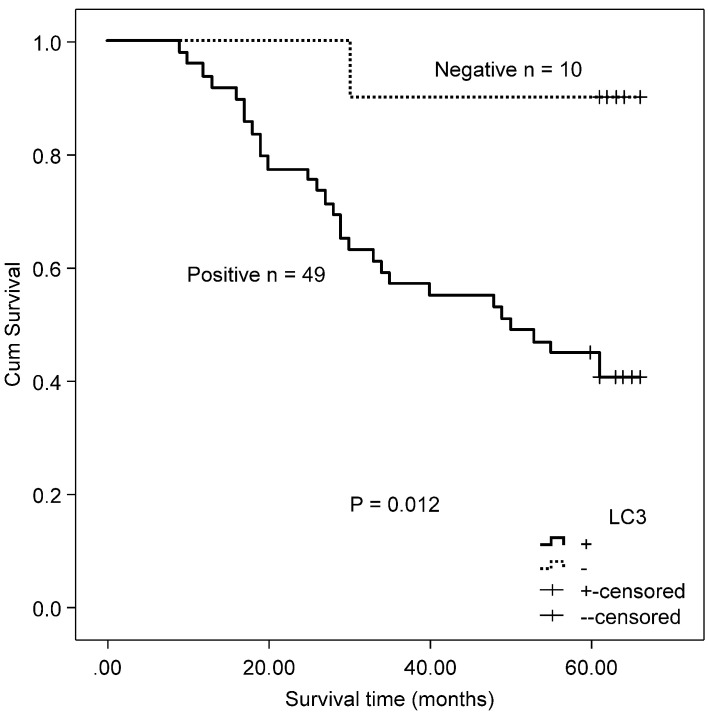
Kaplan-Meier survival curves comparing survival time for CRC patients with mutated *KRAS* expressing LC3.

There was no significant correlation observed between autophagy-related proteins and KRAS by χ^2^ test (Beclin 1: χ^2^ = 0.223, P = 0.637; LC3: χ^2^ = 0.257_,_ P = 0.341, Table 2) and the Spearman's rank test (Beclin 1: r = ‑0.033, P = 0.639; LC3: r = ‑0.026, P = 0.614). Both the protein expression of Beclin 1 and LC3 were unrelated to all clinicopathological parameters in the mutated *KRAS* gene subgroup. Accordingly, no association was found between the protein expression of Beclin 1 and OS in the mutated *KRAS* gene subgroup (χ^2^ = 0.429, P = 0.513). Patients with positive protein expression of LC3 exhibited a poorer outcome compared with patients with negative protein expression of LC3 in the mutated *KRAS* subgroup (χ^2^ = 6.330, P = 0.012, Fig. 4).

The expression of Beclin 1 was demonstrated to be unrelated to all clinicopathological parameters (Table 5). The expression of LC3 was related to TNM stage III/IV (P = 0.014), whereas it was unrelated to other clinicopathological parameters in the subgroup of patients with CRC with wild type *KRAS* (Table 5). No significant association was found between autophagy-related proteins (Beclin 1 and LC3) and OS in the CRC patient subgroup with wild type *KRAS* (Beclin 1: χ^2^ = 2.800, P = 0.094; and LC3: χ^2^ = 2.362, P = 0.124).

### Correlation of autophagy-related proteins (Beclin 1 and LC3) and OS in MSS/L colorectal cancer with mutated/wild type *KRAS*

**Figure 5:**
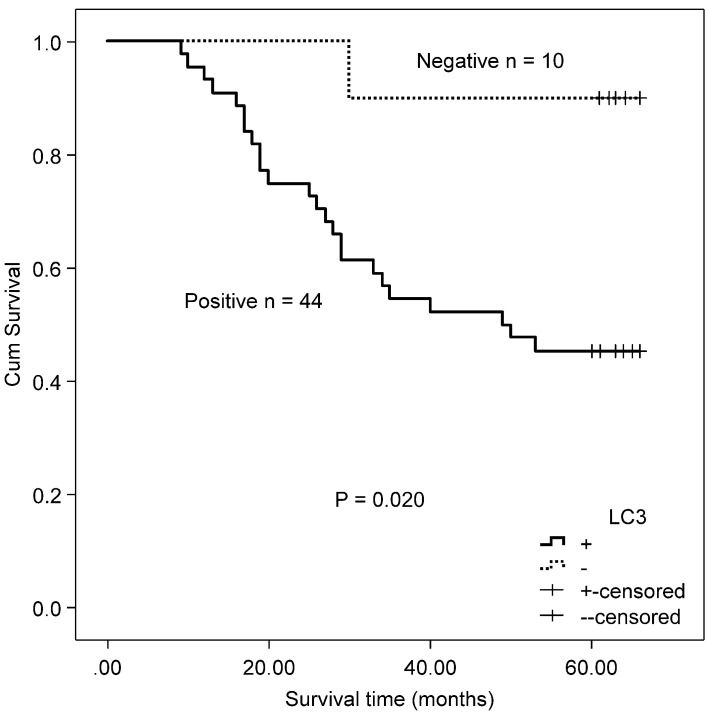
Kaplan-Meier survival curves comparing survival time for MSS/L-CRC patients with mutated *KRAS* with LC3 expression.

A total of 118 patients with MSS/L colorectal cancer, including 54 patients with *KRAS* mutations and 64 *KRAS* wild type patients, were included in this study. The MSS/L-CRC patients bearing *KRAS* mutations positively expressed LC3 protein and suffered a shorter OS than LC3 non-expressing patients (χ^2^ = 5.402, P = 0.020, Fig 5). There was no significant association between Beclin 1 and OS in MSS/L-CRC patients with *KRAS* mutations (χ^2^ = 0.245, P = 0.620). No association between autophagy-related proteins (Beclin 1 and LC3) and OS was found in the group of MSS/L-CRC patients with wild type *KRAS* (Beclin 1: χ^2^ = 2.969, P = 0.085; and LC3: χ^2^ = 1.762, P = 0.184).

### Correlation of autophagy-related proteins (Beclin 1 and LC3) and OS in CRC patients received either capecitabine or capecitabine combined with oxaliplatin after surgery

**Figure 6:**
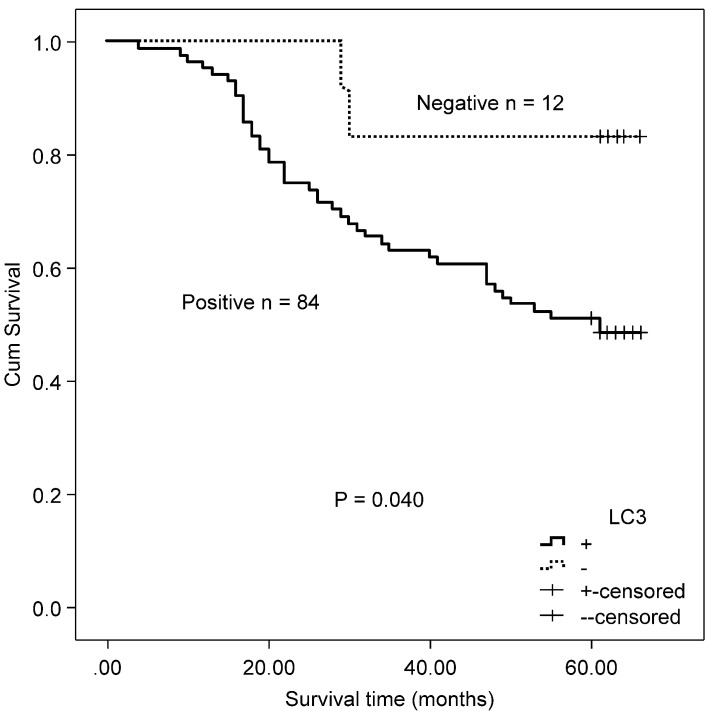
Kaplan-Meier survival curves comparing survival time for LC3 expression with CRC patients who received either capecitabine or capecitabine combined with oxaliplatin after surgery.

Twenty-four patients received capecitabine adjuvant therapy after surgery, and 72 patients received capecitabine combined with oxaliplatin chemotherapy after surgery. There was no difference in the expressions of autophagy-related proteins (Beclin 1 and LC3) between the two therapeutic groups (Beclin 1: χ^2^ = 0.933, P = 0.334; and LC3: χ^2^ = 261, P = 0.609). In CRC patients who received either capecitabine or capecitabine combined with oxaliplatin post-surgery, positive LC3 expression correlated with worse OS relative to non-expressing patients (χ^2^ = 4.216, P = 0.040, Fig. 6). No association was found between Beclin 1 protein expression and OS in these patients (χ^2^ = 2.084, P = 0.149).

### Sequencing results of 19 genes in CRC

**Figure 7:**
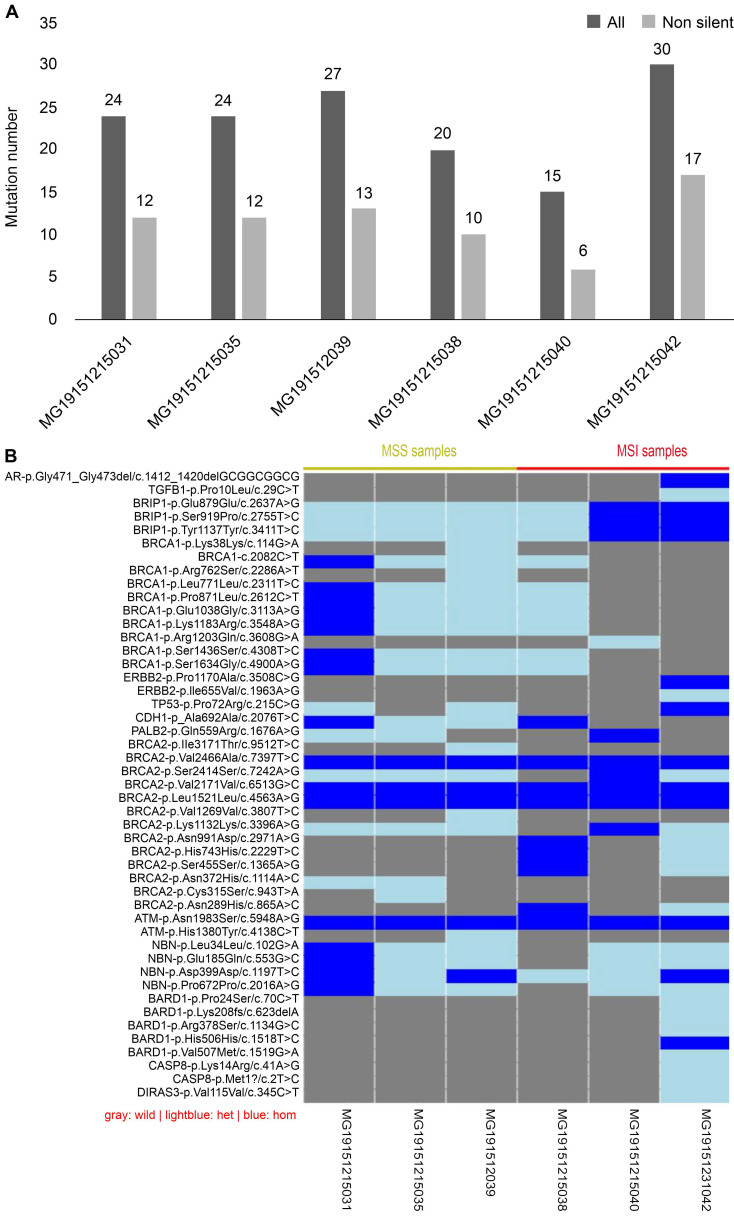
Results of next-generation sequencing in 6 cases CRC samples. (A) Comparison of variants among samples: gray is the number of all variants, while black is the number of variants that do not contain synonymous mutations. (B) Information of the genetic variation of all samples: blue represents a homozygous variation, light blue represents a heterozygous variation, and gray represents no variation in the sample.

Mismatch repair (MMR), which belongs to the DNA repair systems, plays a crucial role in maintaining genome stability 16. MSI has been reported to be caused by mutations in MMR 17. The exon regions of 19 genes, including those involved in the DNA repair systems of genes, tumor suppressor genes, and common genetic markers in tumors, were captured by next-generation sequencing. Forty-seven mutation sites were found in six CRC tissues, including two deletions and 45 single nucleotide polymorphisms (SNP). Of the 47 mutation sites, 44 were known, with 26 being missense mutation sites. Each sample had a different number of variations (Fig. 7A). Compared with MSI samples, the difference in the variations in MSS samples was demonstrated to be smaller, whether containing synonymous mutations or not. Because each sample had a different gene mutation status, we show the variations in all samples one by one in Fig. 7B. For example, *BARD1* p.Lys2208fs is a frameshift mutation, whereas *CASP8* p.Met1 is a nonsense mutation. Both are known to be high-risk variations and were demonstrated to occur in MSI-H samples. Although all intersample variations were mainly concentrated on the *BRCA1* and *BRCA2* genes among the 19 DNA damage repair genes, these variations were shown not to be pathogenic factors.

## Discussion

Autophagy is known to be involved in tumor cell survival and growth 18, 19. In the present study, we investigated the protein expression and clinical significance of two autophagy-related proteins, namely Beclin 1 and LC3, in CRC by IHC. Consistent with literature reports 20, 21, we found both the protein expression of Beclin 1 and LC3 to be significantly higher in CRC tissues than in normal counterpart tissues. Wu et al. showed that LC3 expression was associated with Beclin 1 by Spearman analysis; however, we did not find a significant correlation 20. They also reported that the protein expression of Beclin 1 was not significantly associated with clinicopathological parameters, including patient age, gender, tumor size, primary site, tumor differentiation, TNM stage, and lymph node metastasis 20. Our results were consistent with those observations. We found that the positive expression of Beclin 1 significantly correlated with the depth of invasion, and the correlation was more robust in the T3/T4 group. In contrast, Schmitz et al. reported that the positive expression of Beclin 1 was not associated with the depth of invasion 22. On the other hand, one study showed that the LC3 protein overexpression was associated with reduced cell differentiation and lymph node metastasis 20. In contrast, another study showed that the overexpression of the LC3 protein was not associated with patient sex, age, depth of invasion, TNM stage, and lymph node metastasis 21. In the present study, LC3 expression was demonstrated not to be associated with any assessed clinicopathological parameters. Kaplan-Meier survival analysis of LC3 expressing-patients indicated a worse OS compared to non-expressing patients who underwent postoperative adjuvant therapy, whereas no association was found between the expression of the Beclin 1 protein and OS. Koustas et al. revealed that patients with CRC with low expression levels of the Beclin 1 protein experienced a better OS than patients with high expression levels, whereas no association was found between the expression of the LC3 protein and OS 21. In contrast, Wu et al. came to an opposing finding and reported that the high protein expression of Beclin 1 and LC3 was positively associated with patients' prolonged survival, assuming that they might act as tumor suppressors 20. Due to the insufficient sample size, each study's results differed to some extent, so it is necessary to expand the CRC samples in future studies to analyze further the relationship between the expression of autophagy-related proteins, clinicopathologic features, and prognosis.

It has been reported that MSI is identified in ~15% of patients with CRC 23. In good accordance, the present study results showed that the rate of MSI in patients with CRC was 13%. The MSI-H state was related to age ≤ 50, without lymph node metastasis, located in the right hemicolon, poor tumor differentiation, but not to gender, mucous adenocarcinoma, depth of invasion, and TNM stage. Samowitz et al. reported that MSI-H incidence was high in patients < 50 or > 70 y old, with individuals between these ages exhibiting a low incidence 3. Besides, certain studies have suggested that MSI-H might not be age-related 24-26. The incidence of MSI-H in patients with CRC with age ≤ 50 was higher than that in patients > 50 in this study, in accordance with the findings by Samowitz et al. It has been reported that the incidence of MSI-H in female patients was higher than that in male patients 3, 27, but this and other studies did not reach the same conclusion 26. We agreed with the conclusions of many studies that MSI-H has a high incidence of cancer of the proximal colon in patients with CRC 25, 28-30. A study showed that MSI-H was more common in patients with CRC without lymph node metastasis than in those with lymph node metastasis 28, quite similar to our results. Respectively, studies have shown a high incidence of MSI-H in mucinous colorectal adenocarcinoma 30, which was not confirmed in this study.

Some studies have indicated that MSI-H might still be a better factor for CRC patients' survival 31, 32. However, several other studies came to opposite conclusions, stating that MSI-H might function as an adverse factor for OS in CRC 33. Although the survival curve of patients with MSI-H-CRC was higher than that of patients with MSS/L, because of P > 0.05, we could not assume whether MSI-H might be a beneficial factor in CRC.

5-fluorouracil (5-Fu)-based chemotherapy drugs are currently the first-line chemotherapeutic agents for treating colorectal cancer. Nevertheless, the guidelines of NCCN state that MSI-H CRC patients do not benefit from 5-FU adjuvant therapy 34. Sena et al. reported that the expression of LC3 in MSS cancer cells was higher than that in MSI cancer cells 35; however, we did not identify any difference in the expression of autophagy-related proteins between MSH and MSS/L-CRC patients. Since similar reports are rare, more research is needed to corroborate this result. In addition, there have been no reports on the characteristics of autophagy expression in CRC patients with different microsatellite states. In this study, CRC patients were further divided into MSI-H and MSS/L subgroups, and the relationships between the autophagy-related proteins (Beclin 1 and LC3) and clinicopathological features and prognosis in these two subgroups were also analyzed. We found that the expression of Beclin 1 and LC3 was unrelated to all clinicopathological parameters and OS in the MSI-H-CRC subgroup. However, the sample size of patients in this subgroup is small, and future studies with a larger sample size are warranted. The expression of Beclin 1 was demonstrated to be higher in the T3/T4 group; there was no significant association observed between the expression of the LC3 protein and all clinicopathological parameters in the MSS/L-CRC subgroup. Patients with positive expression of LC3 had a worse OS than those with a negative expression in the MSS/L-CRC subgroup, suggesting that LC3 might be a poor prognostic factor in patients with MSS/L-CRC.

5-FU, combined with other agents such as oxaliplatin, has been shown to improve OS in patients with advanced CRC 36. However, drug toxicity, resistance, and disease relapse are still the most common treatment challenges. Therefore, improved CRC therapeutic efficacy and increased tumor killing are critical to managing patients with CRC. The inhibition of autophagy could increase 5-FU-induced apoptosis in animal experiments 12, 13. In the present research, CRC patients received either capecitabine (a 5-FU chemotherapy drug) or capecitabine combined with oxaliplatin after surgery, LC3 expression correlated with worse OS compared to patients non-expressing LC3, demonstrating that, in CRC patients, an elevated expression of LC3 protein may affect the efficacy of capecitabine-related chemotherapy. LC3 protein expression should be detected when patients opt for capecitabine-related treatment regimens. If patients have high levels of LC3 protein, other chemotherapy regimens including autophagy inhibitors are recommended to be part of their treatment strategy.

The percentage of mutations in the *KRAS* gene in CRC is known to be 35-45% 37. In this study, the incidence of mutation in patients with CRC was reported to be 40%, consistent with most reports. Many studies have examined the relationships between *KRAS* mutations with various clinicopathologic characteristics, however, with no consistent results 38-44. We found that the incidence of mutations in the *KRAS* gene was related to gender, depth of invasion, and TNM stage in CRC, but not related to age, lymph node metastasis, tumor location, tumor differentiation, or mucous adenocarcinoma. Li et al. reported that the rate of mutations in *KRAS* was different in primary tumor sites, genders, and tumor histology types 38. Kadowaki et al. also found that mutations in *KRAS* were associated with gender but with no other variables 41. Niu et al. reported that mutations in *KRAS* in patients with stage III CRC were related to the proximal colon and pathological stage but not related to sex, age, lymph node metastasis, or infiltration depth 39. Such inconsistencies might have arisen due to differences in the distribution of race, age, stage, or other factors in the study population. At present, no convincing evidence has demonstrated that mutations in *KRAS* might have an independent prognostic role in CRC. Previous studies indicated that patients with CRC with mutations in *KRAS* had a significantly increased risk of death or recurrence compared with those with a wild type *KRAS* 38, 40, 41. However, no association was identified between mutations in the *KRAS* gene and OS in patients with CRC in the current research and in agreement with Liou et al. 42.

Previus studies have demonstrated that mutations in *KRAS* can directly lead to a failure for CRC patients subjected to anti-epidermal growth factor receptor (EGFR) therapy, such as cetuximab 45, 46. Moreover, it has been reported that the effect of cetuximab on colon cancer cells might be improved by autophagy 47. At present, the relationship between autophagy and KRAS in colorectal cancer remains obscure. We investigated the relationships between mutations in *KRAS* and autophagy-related proteins in patients with CRC and did not identify a significant correlation. It has not been reported that autophagy characteristics in CRC patients carry or not (wild type) mutations in *KRAS* at present*.* We first reported that Beclin 1 was unrelated to all clinicopathological parameters in patients with CRC with wild type* KRAS*, whereas the expression of LC3 was related to TNM stage III/IV in the same subgroup. The expression of Beclin 1 and LC3 proteins was also first shown to be unrelated to all clinicopathological parameters in the mutated KRAS gene subgroup in the current paper. A previous study on CRCs reported that patients with Beclin 1 nuclear (not cytoplasmic) staining had a significantly decreased OS in the only 34 cases of *KRAS*-mutated CRC patients and did not find that in the wild type *KRAS* CRCs 22. Our study included a larger number of patients; in disagreement with previous findings, we observed that the reported associations between Beclin 1 protein expression and OS were absent in both mutated and wild type *KRAS* subgroups. This study reported positive nuclear staining of the Beclin 1 protein; however, we mainly observed a cytoplasmic staining pattern in our study. It needs to conduct additional research focus on the cellular localizations about Beclin 1 protein. They also found that overexpression of LC3 was significantly related to worse OS in the *KRAS*-mutated CRC group, which was not found in the wild type *KRAS* CRC group 22, consistent with our findings. We gave a novel finding that CRC patients with different KRAS gene states, different autophagy markers may play different prognostic values. A larger sample size of stratified analysis should be examined to confirm these results. For CRC patients with different KRAS gene states, research schemes of targeted therapy for autophagy may be different.

As per NCCN guidelines, RAS and MSI are important molecular features in patients with colorectal cancer and should be considered while tailoring the ideal therapeutic approach for their clinical management 48. We too believe that status of autophagy, RAS, and MSI should be analyzed in combination in patients with CRC. However, in this study, no association was observed between autophagy-related proteins and OS in MSS/L-CRC patients with wild type *KRAS*. This finding suggests that for these patients, the detection of autophagy-related protein may be unnecessary in clinical practice; this would also reduce patients' economic burden. Of note, LC3 protein expressing MSS/L-CRC patients with *KRAS* mutations have a shorter OS than LC3 non-expressing patients. Thus, patients, who have undergone chemotherapy, should also be treated with autophagy inhibitors or other treatments, such as immunotherapy or radiation therapy. Further clinical trials are warranted to investigate the efficacy of these therapeutic strategies.

Next-generation sequencing was used in this study to capture the information on the variation of 19 genes to explore their relationship with MSI. Lin et al. found that the mutation number of MSI-H CRCs was significantly higher than MSS CRCs. Compared with MSI samples, the difference in MSS intersample variation was demonstrated to be smaller whether containing synonymous mutations or not. However, due to the small sample size, the difference in the number of variations between the two groups could not be compared. We found that all the variations in the samples were mainly concentrated on the *BRCA1* and *BRCA2* genes, but when compared with data obtained from a thousand-genome database, these variations were shown not to be pathogenic. We first identified the *BARD1* p.Lys2208fs frameshift mutation and the *CASP8* p.Met1 nonsense mutation, which were only observed on MSI-H patients. The BARD1 gene has been reported to be often upregulated and associated with worse outcomes in various tumors, such as breast, ovarian, endometrial, and lung cancers 49, 50. However, its tumorigenic mechanism has not been reported in colorectal cancer. In addition, several *CASP8* gene SNPs are reportedly associated with various types of cancer 51. However, the CASP8 p.Met1 nonsense mutation has not been previously identified in colorectal cancer. To verify whether this mutation might be incidental in colorectal cancer or not, or whether it might be associated with colon cancer or MSI, more specimens are needed.

## Conclusion

In summary, the expression of autophagy-related proteins (Beclin 1 and LC3) was elevated in CRC tissues. LC3-expressing patients who underwent adjuvant therapy experienced a shorter OS after surgery, especially in the MSS/L-CRC and mutated *KRAS* subgroups. MSS/L-CRC patients with *KRAS* mutation positively expressed LC3 protein and suffered a shorter OS than LC3 non-expressing patients. LC3 is suggested as a recommendable novel prognostic marker to personalize treatment in CRC patients that do not respond to chemotherapy. LC3, MSI status, and *KRAS* mutations are critical factors that may affect the efficacy of adjuvant therapy during CRC chemotherapy. The detection of these indexes is of great significance to select ideal clinical treatments for patients with CRC. Nevertheless, further clinical trials are needed to investigate therapeutic strategies combined with autophagy inhibitors, immunotherapy, or radiation therapy.

## Supplementary Material

Supplementary figures and tables.

## Figures and Tables

**Table 1:** The expression of autophagy-related proteins (Beclin 1 and LC3) in the colorectal cancer.

Score	Beclin1		t	P	χ^2^	P	LC3		t	P	χ^2^	P
	tumor samples n (%)	intestinal mucosa n (%)					tumor samples n (%)	intestinal mucosa n (%)				
9	107(53.5)	2(1.0)	28.069	<0.001	274.337	<0.001	95(47.5)	4(2.0)	30.066	<0.001	281.999	<0.001
6	61(30.5)	2(1.0)					78(39.0)	2(1.0)				
3	8(4.0)	11(5.5)					11(5.5)	10(5.0)				
0	24(12.0)	185(92.5)					16(8.0)	184(92.0)				

**Table 2:** Association between the expression of autophagy-related proteins Beclin 1 and LC3 and clinicopathologic features of colorectal cancer.

Variable	n		Beclin 1			LC3	
		+ (%)	-(%)	P	+ (%)	-(%)	P
Gender				0.587			0.442
Male	110	91 (82.72)	19 (17.28)		97 (88.18)	13 (11.82)	
Female	90	77 (85.56)	13 (14.44)		76 (84.44)	14 (15.56)	
Age (y)				0.852			0.661
≤ 50	59	50 (84.75)	9 (15.25)		52 (88.14)	7 (11.86)	
> 50	141	118 (83.69)	23 (16.31)		121 (85.82)	20 (14.18)	
Lymph node metastasis				0.083			0.327
No	116	93 (80.17)	23 (19.83)		98 (84.48)	18 (15.52)	
Yes	84	75 (89.28)	9 (10.72)		75 (89.28)	9 (10.72)	
Location				0.852			0.356
Right hemicolon	59	50 (84.75)	9 (15.25)		49 (83.05)	10 (16.95)	
Left hemicolon	141	118 (83.69)	23 (16.31)		124 (87.94)	17 (12.06)	
Tumor differentiation				0.887			0.778
Poor	79	66 (83.54)	13 (16.46)		69 (87.34)	10 (12.66)	
Moderate/well	121	102 (84.30)	19 (15.70)		104 (85.95)	17 (14.05)	
Mucous adenocarcinoma				0.703			0.846
No	164	137 (83.54)	27 (16.46)		141 (85.98)	23 (14.02)	
Yes	36	31 (86.11)	5 (13.89)		32 (88.89)	4 (11.11)	
Depth of invasion				0.005^*^			0.401
T1/T2	30	20 (66.67)	10 (33.33)		24 (80.00)	6 (20.00)	
T3/T4	170	148 (87.06)	22 (12.94)		149 (87.65)	21 (12.35)	
TNM stage				0.340			0.327
I/II	116	95 (81.90)	21 (18.10)		98 (84.48)	18 (15.52)	
III/IV	84	73 (86.90)	11 (13.10)		75 (89.29)	9 (10.71)	
MSI				0.341			0.341
MSI-H	26	24 (92.31)	2 (7.69)		21 (80.76)	5 (19.24)	
MSS/L	174	144 (82.75)	30 (17.25)		152 (87.36)	22 (12.64)	
KRAS mutation status				0.637			0.341
Mutated type	80	66 (82.50)	14 (17.50)		68 (85.00)	12 (15.00)	
Wild type	120	102 (85.00)	18 (15.00)		105 (87.50)	15 (12.50)	

*P < 0.05 indicates a significant association among the variables. MSI, microsatellite instability; MSI-H, high frequency microsatellite instability; MSI-L, low frequency microsatellite instability; MSS, microsatellite stable.

**Table 3:** Association between MSI, *KRAS* mutations*,* and clinicopathologic features of colorectal cancer.

Variable	Cases	MSI			KRAS		
		MSI-H (%)	MSS/L (%)	P	Mutation type (%)	Wild type (%)	P
Gender				0.676			<0.001^*^
Male	110	15 (13.64)	95 (86.36)		32 (29.09)	78 (70.91)	
Female	90	11 (12.22)	79 (87.78)		48 (53.33)	42 (46.67)	
Age (y)				0.046^*^			0.899
≤50	59	12 (20.34)	47 (79.66)		24 (40.68)	35 (59.32)	
>50	141	14 (9.92)	127 (90.08)		56 (39.72)	85 (60.28)	
Lymph node metastasis				0.036^*^			0.061
No	116	20 (17.24)	96 (82.76)		40 (34.48)	76 (65.52)	
Yes	84	6 (7.14)	78 (92.86)		40 (47.62)	44 (52.38)	
Location				<0.001^*^			0.411
Right hemicolon	59	17 (28.81)	42 (71.19)		21 (35.59)	38 (64.41)	
Left hemicolon	141	9 (6.38)	132 (93.62)		59 (41.84)	82 (58.16)	
Tumor differentiation				0.004^*^			0.906
Poor	79	17 (21.52)	62 (78.48)		32 (40.50)	47 (59.50)	
Moderate/well	121	9 (7.44)	112 (92.56)		48 (39.67)	73 (60.33)	
Mucous adenocarcinoma				0.204			0.548
No	164	19 (11.56)	145 (88.46)		64 (39.02)	100 (60.97)	
Yes	36	7 (19.44)	29 (80.56)		16 (44.44)	20 (5.56)	
Depth of invasion				0.158			0.043^*^
T1/T2	30	1 (3.33)	29 (96.67)		17 (56.67)	13 (43.33)	
T3/T4	170	25 (14.71)	145 (85.29)		63 (37.06)	107 (62.94)	
TNM stage							
I/II	116	19 (16.38)	97 (83.62)	0.095	38 (32.76)	78 (67.24)	0.014^*^
III/IV	84	7 (8.33)	77 (91.67)		42 (50.00)	42 (50.00)	

*P < 0.05 indicates a significant association among the variables. MSI, microsatellite instability; MSI-H, high frequency microsatellite instability.

**Table 4:** Association between the expression of autophagy-related proteins Beclin 1 and LC3 and clinicopathologic features in patients with colorectal cancer and MSS/L.

Variable	n	Beclin 1			LC3		
		+ (%)	- (%)	P	+ (%)	- (%)	P
Gender				0.802			0.168
Male	95	78 (82.11)	17 (17.89)		86 (90.53)	9 (9.47)	
Female	79	66 (83.54)	13 (16.46)		66 (83.54)	13 (16.46)	
Age (y)				0.963			0.628
≤50	47	39 (82.98)	8 (17.02)		42 (89.36)	5 (10.64)	
>50	127	105 (82.68)	22 (17.32)		110 (86.61)	17 (13.39)	
Lymph node metastasis				0.073			0.693
No	96	75 (78.13)	21 (21.87)		83 (86.46)	13 (13.54)	
Yes	78	69 (88.46)	9 (11.54)		69 (88.46)	9 (11.54)	
Location				0.722			0.368
right hemicolon	42	34 (80.95)	8 (19,05)		35 (83.33)	7 (16.67)	
left hemicolon	132	110 (83.33)	22 (16.67)		117 (88.64)	15 (11.36)	
Tumor differentiation				0.897			0.689
Poor	62	51 (82.26)	11 (17.74)		55 (88.71)	7 (11.29)	
Moderate/well	112	93 (83.04)	19 (16.96)		97 (86.61)	15 (13.39)	
Mucous adenocarcinoma				0.590			0.475
No	145	119 (82.07)	26 (17.93)		125 (86.21)	20 (13.79)	
Yes	29	25 (86.21)	4 (13.79)		27 (93.10)	2 (6.90)	
Depth of invasion				0.031^*^			0.610
T1/T2	29	20 (68.97)	9 (31.03)		24 (82.76)	5 (17.24)	
T3/T4	145	124 (85.52)	21 (14.48)		128 (88.28)	17 (11.72)	
TNM stage				0.358			0.735
I/II	97	78 (80.41)	19 (19.59)		84 (86.60)	13 (13.40)	
III/IV	77	66 (85.71)	11 (14.29)		68 (88.31)	9 (11.69)	

*P < 0.05 indicates a significant association between variables. MSI, microsatellite instability; MSI-L, low frequency microsatellite instability; MSS, microsatellite stable.

**Table 5:** Association between the expression of autophagy-related proteins Beclin 1 and LC3 and clinicopathologic features in patients with colorectal cancer and wild type *KRAS.*

Variable	n		Beclin 1			LC3	
		+ (%)	- (%)	P	+ (%)	- (%)	P
Gender				0.486			0.311
Male	78	65 (83.33)	13 (16.67)		70 (89.74)	8 (10.26)	
Female	42	37 (88.10)	5 (11.90)		35 (83.33)	7 (16.67)	
Age (y)				0.673			0.595
≤ 50	35	29 (82.86)	6 (17.14)		32 (91.43)	3 (8.57)	
> 50	85	73 (85.88)	12 (14.12)		73 (85.88)	12 (14.11)	
Lymph node metastasis				0.396			0.152
No	76	63 (82.89)	13 (17.11)		64 (84.21)	12 (15.79)	
Yes	44	39 (88.64)	5 (11.36)		41 (93.18)	3 (6.82)	
Location				0.869			0.656
right hemicolon	38	32 (84.21)	6 (15.79)		32 (84.21)	6 (15.79)	
left hemicolon	82	70 (85.37)	12 (14.63)		73 (89.02)	9 (10.98)	
Tumor differentiation				0.979			0.104
Poor	47	40 (85.11)	7 (14.89)		44 (93.62)	3 (6.38)	
Moderate/well	73	62 (84.93)	11 (15.07)		61 (83.56)	12 (16.43)	
Mucous adenocarcinoma				1.000			1.000
No	100	85 (85.00)	15 (15.00)		87 (87.00)	13 (13.00)	
Yes	20	17 (85.00)	3 (15.00)		18 (90.00)	2 (10.00)	
Depth of invasion				0.202			0.912
T1/T2	13	9 (69.23)	4 (30.77)		12 (92.31)	1 (7.69)	
T3/T4	107	93 (86.92)	14 (13.08)		93 (86.92)	14 (13.08)	
TNM stage				0.872			
I/II	78	66 (84.62)	12 (15.38)		64 (82.05)	14 (17.95)	0.014*
III/IV	42	36 (85.71)	6 (14.29)		41 (97.62)	1 (2.38)	

*P < 0.05 indicates a significant association between variables.

## Abbreviations

CRC: Colorectal cancer
MSI: Microsatellite instability
CIN: Chromosomal instability
MSI-H: High-frequency microsatellite instability
MSI-L: Low-frequency microsatellite instability
EFFR: Epidermal growth factor receptor
ATG: Autophagy-related genes
BARD1: BRCA1 associated RING domain 1
DIRAS3: DIRAS family GTPase 3
ERBB2: Erb-B2 receptor tyrosine kinase 2
NBN: Nibrin
PALB2: Partner and localizer of BRCA2
PTEN: Phosphatase and tensin homolog
TGFB1: Transforming growth factor-beta 1
TP53: Tumor protein p53
