# Supplementary material for: Prognostic Value of Autophagy, Microsatellite Instability, and KRAS Mutations in Colorectal Cancer
Source: J Cancer. 2021 Apr 20;12(12):3515–28. doi: 10.7150/jca.51430 (PMC8120188; doi:10.7150/jca.51430)
Supplement: Supplementary file 1 — Supplementary figures and tables. [file jcav12p3515s1.pdf]

Supplementary Material

Supplementary Table 1. MSI status in CRC.

MSI state	n	Bat26	Bat25	D5S346	D2S123	D17S250
MSI-H	11	+	+	-	-	-
	2	+	+	+	-	-
	3	+	+	-	+	-
	5	+	+	-	+	+
	2	+	+	-	-	+
	3	+	+	-	-	+
MSI-L	1	-	+	-	-	-
MSS	173	-	-	-	-	-

Supplementary Table 2. *KRAS* mutations in CRC.

Detected region	Mutation	Base change	Mutation cases	Mutation rate (%)
Codon 12	G12D	35G > A	33	41.25
	G12V	35G > T	18	22.50
	G12A	35G > C	7	8.75
	G12C	34G > T	4	5.00
	G12S	34G > A	3	3.75
Codon 13	G13D	38G > A	15	18.75

CRC, colorectal cancer

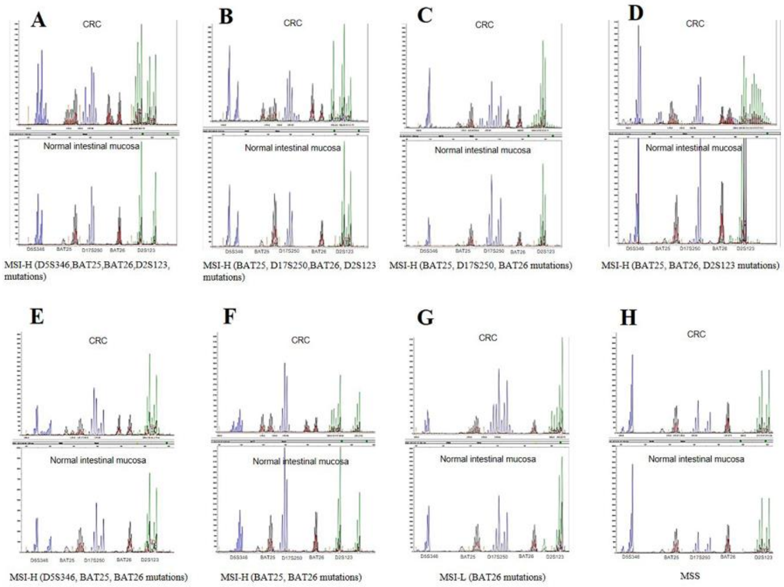

Supplement figure 1. The gold standard for MSI testing recommended by the National Cancer Institute is using polymerase chain reaction to detect MSI markers (BAT25, BAT26, D2S123, D5S346, and D17S250). High-frequency microsatellite instability (MSI-H) with two or more genes showing instability; low-frequency microsatellite instability (MSI-L) with only one locus showing genetic deletion; microsatellite stable (MSS) CRC with no gene loss. (A–F) MSI-H; (G) MSI-L; (H) MSS.

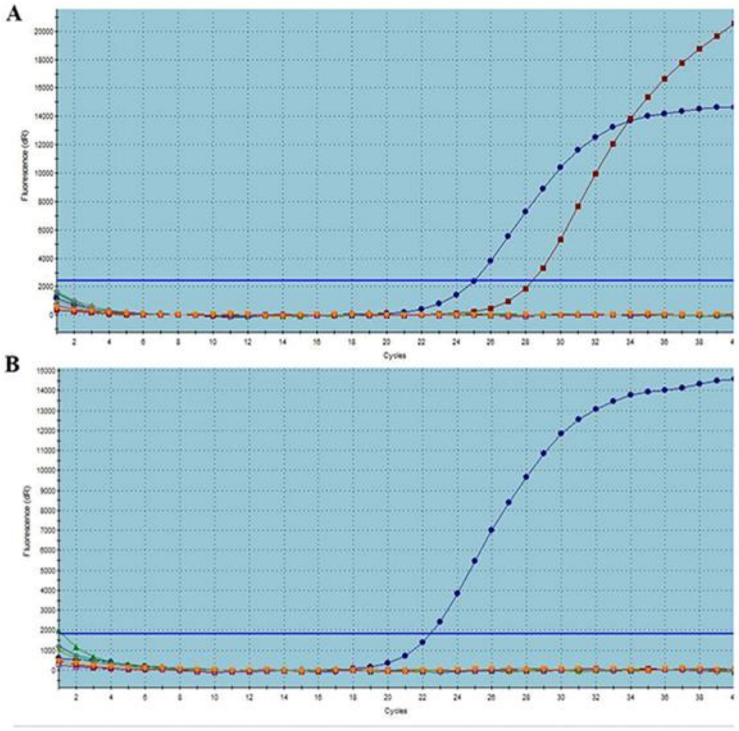

Supplement figure 2. (A) KRAS mutation type; (B) KRAS wild type. The blue line represents GAPDH as a reference gene, whereas the red line representing the *KRAS* mutation curve.
